# Facial emotion recognition deficits in first-degree relatives of patients with bipolar disorder: a systematic review protocol

**DOI:** 10.1192/j.eurpsy.2022.1960

**Published:** 2022-09-01

**Authors:** C. Calçada, A.R. Ferreira, L. Fernandes

**Affiliations:** 1 Faculty of Medicine, University Of Porto, Porto, Portugal; 2 Faculty of Medicine, University of Porto, Department Of Clinical Neurosciences And Mental Health, Porto, Portugal; 3 Faculty of Medicine, University of Porto, Cintesis – Center For Health Technology And Services Research, Porto, Portugal; 4 Psychiatry Service, Centro Hospitalar Universitário De São João, Porto, Portugal

**Keywords:** Facial Emotion Recognition deficits, First-degree relatives, systematic review, bipolar disorder

## Abstract

**Introduction:**

Bipolar Disorder (BD) is one of the most challenging and severe psychiatric disorders. Considerable research in BD patients points to deficits in Facial Emotion Recognition (FER) as a potential BD endophenotype. Accordingly, such deficits have also been found in unaffected BD first-degree relatives, but no study has been conducted to synthetize this evidence.

**Objectives:**

To conduct a systematic review of studies exploring FER deficits in first-degree relatives of patients with BD.

**Methods:**

PRISMA 2020 recommendations will be followed. PubMed, Scopus, Web of Science and SciELO electronic bibliographic databases will be searched, as well as grey literature. Reference lists of the included studies will be hand-searched for additional eligible studies. Search strategy will include key-terms in accordance with the pre-established PICOS definition. No restrictions will apply regarding study design, setting, publication date nor language. Outcomes of interest will be FER deficits. Retrieved studies will be screened for eligibility by two independent reviewers using a two-phase approach. The methodological quality of primary studies will be assessed and data extracted independently using a standardized extraction form.

**Results:**

will be described using narrative and tabular approaches. Studies heterogeneity will be verified and if adequate a meta-analysis will be conducted. Findings will be disseminated through a peer-reviewed publication.

**Conclusions:**

It is expected that this systematic review will support the hypothesis that FER deficits may constitute a potential candidate for a BD endophenotype, which will not only improve the understanding of BD neurobiology, but also enable its identification in earlier stages, allowing timely treatments and better patients’ outcomes.

**Disclosure:**

No significant relationships.

